# Development of Heteroatomic Constant Potential Method with Application to MXene-Based Supercapacitors

**DOI:** 10.1021/acs.jctc.3c00940

**Published:** 2024-01-11

**Authors:** Xiaobo Lin, Shern R. Tee, Paul R. C. Kent, Debra J. Searles, Peter T. Cummings

**Affiliations:** † Multiscale Modeling and Simulation Center, Vanderbilt University, Nashville, Tennessee 37235-1604, United States; ‡ Department of Chemical and Biomolecular Engineering, Vanderbilt University, Nashville, Tennessee 37235-1604, United States; § Australian Institute for Bioengineering and Nanotechnology, The University of Queensland, Brisbane, QLD 4072, Australia; ∥ Computational Sciences and Engineering Division, Oak Ridge National Laboratory, Oak Ridge, Tennessee 37830, United States; ⊥ School of Chemistry and Molecular Biosciences, The University of Queensland, Brisbane, QLD 4072, Australia; # School of Engineering and Physical Sciences, Heriot-Watt University, Edinburgh, Scotland EH14 4AS, U.K.

## Abstract

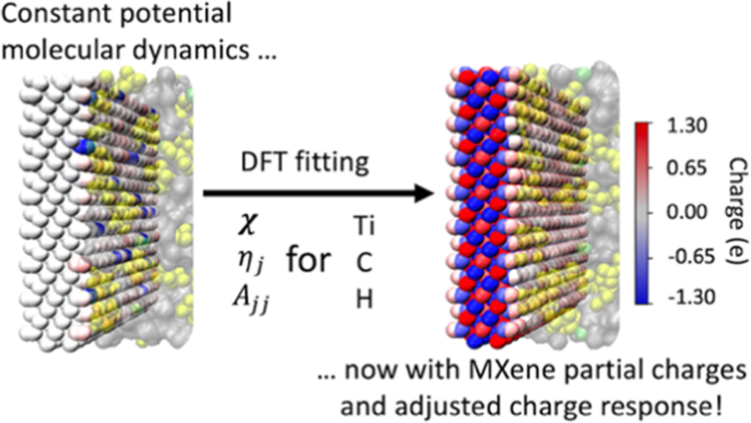

We describe a method for modeling constant-potential charges in heteroatomic electrodes, keeping pace with the increasing complexity of electrode composition and nanostructure in electrochemical research. The proposed “heteroatomic constant potential method” (HCPM) uses minimal added parameters to handle differing electronegativities and chemical hardnesses of different elements, which we fit to density functional theory (DFT) partial charge predictions in this paper by using derivative-free optimization. To demonstrate the model, we performed molecular dynamics simulations using both HCPM and conventional constant potential method (CPM) for MXene electrodes with Li-TFSI/AN (lithium bis(trifluoromethane sulfonyl)imide/acetonitrile)-based solvent-in-salt electrolytes. Although the two methods show similar accumulated charge storage on the electrodes, the results indicated that HCPM provides a more reliable depiction of electrode atom charge distribution and charge response compared with CPM, accompanied by increased cationic attraction to the MXene surface. These results highlight the influence of elemental composition on electrode performance, and the flexibility of our HCPM opens up new avenues for studying the performance of diverse heteroatomic electrodes including other types of MXenes, two-dimensional materials, metal–organic frameworks (MOFs), and doped carbonaceous electrodes.

## 1 Introduction

Supercapacitors are a promising class of electrochemical energy storage devices that can either supplement or be used as an alternative to batteries, especially in applications where fast energy storage and high power density are required.^1^ However, supercapacitors generally have lower energy density than batteries, making the design and discovery of novel electrode and electrolyte materials crucial for enhancing their performance.^2^

MXenes, a family of two-dimensional (2D) transition metal carbides and carbonitrides, have garnered significant attention as electrode materials for use in supercapacitors and electrocatalysis.^3−9^ MXenes have the chemical formula M_*n*–1_X*_n_*T_*x*_, where M is a transition metal, X stands for carbon or nitrogen, and T represents the surface termination groups (=O, −OH, −F). Ti_3_C_2_T_*x*_-based supercapacitors have exhibited high Young’s modulus, large specific surface area, high electrical conductivity, volumetric capacitance, and excellent cycle life.^7,8,10−13^ The modification of MXene surface groups also introduces unique properties to MXene and has been found to enhance charging dynamics.^14,15^ Meanwhile, highly concentrated salt solutions—known as solvent-in-salt (SIS) electrolytes—are emerging as promising electrolytes for next-generation high-energy-density supercapacitors or batteries,^16,17^ as they offer expanded voltage windows and enhanced electrochemical stability.^18,19^ Among the solvents being considered for use in SIS electrolytes, acetonitrile (AN) can dramatically enhance the ionic conductivity of electrolytes while maintaining high chemical stability.^19−22^

For decades, molecular dynamics (MD) simulation has been a crucial tool for offering valuable insights into the behavior and mechanism of materials at the molecular level, enabling researchers to further improve the performance of supercapacitors.^23−29^ The utilization of the constant potential method (CPM) in MD simulations is essential for understanding the charging and discharging behavior of supercapacitors.^30^ CPM allows for charge fluctuation on the electrodes so that all atoms in an electrode are constrained to the same electric potential, as expected in a conductor.^31,32^ This approach provides a clear picture of the charge distribution on the electrodes, with the electrode atom charges being updated based on the surrounding environment as the simulation evolves. However, the conventional CPM has limitations when it comes to describing heteroatomic materials, as it does not take into account the different electronegativities and charge localization of the atoms in heteroatomic electrodes, which are composed of different elements.^33^ Despite this simplification, various studies have reported CPM MD simulations of conductive metal–organic framework (MOFs),^24^ amorphous indium gallium zinc oxide (a-IGZO),^34^ molybdenum disulfide (MoS_2_),^35^ and even MXenes.^36^ However, a study by Bi and Salanne^29^ found that incorporating the electronegativity of the electrode atoms in a 1T-MoS_2_ supercapacitor into CPM molecular dynamics simulations is necessary to get a more realistic prediction of the charging mechanism. This raises questions about the impact of the unique properties of different MXene atoms, such as electronegativity and electrode metallicity, on the charge distribution and electrolyte structure of MXene-based supercapacitors.

In this work, we proposed a heteroatomic constant potential method (HCPM) for the study of MXene-based supercapacitors and other heteroatomic conductors. The model introduces a straightforward approach for representing the electronegativity of each element with adjustable parameters for tuning the charge response across the same and different types of atoms. We obtain values for these parameters by utilizing derivative-free optimization to fit the induced charges calculated by density functional theory (DFT). We then compare HCPM and CPM MD simulations and show that HCPM more accurately describes the electrode charge distribution, the interaction of ions with the MXene surface, and ultimately the predicted interfacial structure of the electrolyte, justifying its use in future studies.

## 2 Computational Methods

### 2.1 HCPM Algorithm

CPM MD uses dynamically updated charges to simulate electrodes as conductive objects. At each step, electrode charges are updated to minimize the system’s energy, ensuring that the atoms of each electrode are at equal electrochemical potential.^36^ Although this method is becoming increasingly widespread, it has mostly been used to study carbon-based or metallic electrodes made up of a single element, sidestepping the requirement to model elements of different electronegativity in heteroatomic electrodes. To accurately simulate heteroatomic electrodes, the CPM must place non-neutral partial charges on atoms of different elements even at zero potential. Such nonzero charges would align with both theoretical expectations (that different elements have different electronegativities) and computational results (similar nonzero charges assigned from DFT simulations of electrodes). Over the following subsections, we first summarize the basic CPM method and then describe our minimal extension of CPM into a heteroatomic CPM (HCPM) theory with the following features:

- Electronegativities of different elements are represented with force field partial charges.
- Gaussian charge widths and hardness offsets are customized for different elements, qualitatively capturing charge localization or delocalization effects.
- Between-atom interaction kernels consistently incorporate the width and hardness parameters.
- Width and hardness parameters are fitted to “induced” charges from DFT calculations, that is, the charge difference arising in DFT calculations of the electrode slab with, and without, nearby electrolyte ions, is used as target data for our HCPM model to replicate by tuning the width and hardness parameters.

#### 2.1.1 Summary of the Basic CPM Method and HCPM Additions

Consider a molecular dynamics simulation composed of electrolyte and electrode particles. In this simulation electrolyte particles will have changing positions but constant charge, while electrode particles will have constant positions, but their charges will be updated dynamically as per CPM. In such a simulation, the overall potential energy of the system can be written as
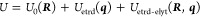
1Here, *U*_0_ is the energy only dependent on electrolyte particle positions, ***R***, but not dependent on electrode charges ***q***, such as non-Coulombic electrode–electrolyte interactions and electrolyte–electrolyte Coulombic interactions. *U*_etrd_ is the electrodes’ self-energy dependent on only electrode charges, while *U*_etrd-elyt_ is the interaction energy between the constant electrolyte charges and variable electrode charges. To determine the charges, we can express the energies *U*_etrd-elyt_ and *U*_etrd_ in terms of ***q***
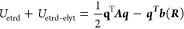
2where the matrix ***A*** depends on the fixed electrode atom positions, and the vector ***b***(***R***) depends on the fixed charges of the electrolyte, the fixed electrode positions, and the variable electrolyte positions. The electrochemical potential on electrode atoms, **Ψ**, is the derivative of overall system energy with respect to the charges on electrode atoms
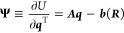
3Then, **Ψ** can be split into three vectors
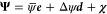
4where ψ̅ is the overall offset potential for electroneutrality,^32^***e*** is a vector with all elements equal to 1, Δψ is the potential difference we set, ***d*** is an indicator vector with entries of 1/2 corresponding to atoms on the positive electrode and −1/2 for atoms on the negative electrode, and **χ** accounts for the electronegativity differences between elements, which have only been included in one other constant potential study so far.^29^ We now consider what happens when **χ** is nonzero to see how force field partial charges can be included in heteroatomic CPM.

#### 2.1.2 Representing Differing Electronegativities in Electrodes with Force Field Charges

The set of charges q* that fixes the potential difference between the two electrodes, while equalizing the potential at each electrode atom on an electrode, maintains electroneutrality and minimizes the energy of electrode charge (according to eq 1) with respect to the charges, and can be written as
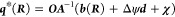
5Here, ***O*** ≡ ***I*****–** (***A***^–1^***ee***^**T**^)/(***e***^**T**^***A***^–1^***e***) is an orthogonalizing matrix that projects the solution q* into the nearest neutral configuration.^32^ Clearly, eq 5 lets us separate q* into two components, one of which is independent of **χ**
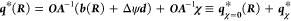
6The second term, ***q***_χ_^*^ = ***OA***^–1^**χ**, does not depend on electrolyte positions and is fixed for immobile electrodes. In the absence of an applied potential or electrolyte, it will give the total charge on the atom. The values of ***q***_χ_^*^ can thus be considered as partial charges which are familiar in any MD simulation implementations. Meanwhile, the first term is the CPM charge calculated in the usual way. Theoretically, this corresponds to using the CPM procedure to calculate a perturbation of the electrode charges because of the presence of the electrolyte.^37^

There are different ways to determine the values of ***q***_χ_^*^. For example, these could be values from existing force fields or results from various charge analysis techniques applied to quantum chemical calculations, or a combination of these choices could be used, as discussed below. The relationship between ***q***_χ_^*^ and **χ** appears different to the recent study by Bi and Salanne^29^ who set ***q***_χ_^*^ = ***A***^–1^**χ**, but is equivalent if the sum of the charges, ***q***_χ_^*^, is zero. However, their parametrization differs, as discussed below.

In addition, in modeling a conducting or semiconducting electrode, the charge density of any atom should be considered, rather than just treating the system as a set of fixed point charges. This will affect the electrode Coulomb interactions through matrix, ***A***
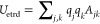
6awhere *j* and *k* refer to different atoms on an electrode. In the original CPM models, this has been considered through the interaction of Gaussian charge distribution centered on each atom
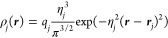
6bwhere *q*_*j*_ is the total charge on atom *j,**r***_*j*_ is the position of the atom *j*, and η_*j*_ determines the width of the distribution of charge about the atom. Then, the total Coulomb interaction energy between electrode atoms can be written as
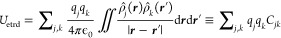
6cwhere ρ̂_*j*_(***r***) = ρ_*j*_(***r***)/*q*_*j*_ and ρ̂_*k*_(***r***′) = ρ_*k*_(***r***′)/*q*_*k*_. Thus, in the original CPM, the interaction between the electrode and electrolyte is given by the interaction kernel ***A*** = ***C***. In addition
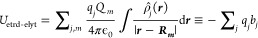
6dwhere *m* refers to an atom in the electrolyte, *Q*_*m*_ is the point charge of atom *m*, ***R***_***m***_ stands for the position of atom *m*, and *b*_*j*_ is the electrostatic potential arising from the electrolyte experienced by atom *j*. This approach assumes a classical model for the Coulomb interactions and that the charge distributions for the partial and induced charges are the same. Determination of the width parameters for each atom in the conducting electrode is still required, and this could be done in various ways such as fitting results of DFT calculations. The value of η_*j*_ is the same for each atom in the original CPM since it was developed for systems where the electrode atoms are all equivalent. In the previous work by Bi and Salanne,^29^ this model is used with the values of η_*j*_ for each atom determined from experimental covalent radii^38^ and the values of ***q***_χ_^*^ determined from quantum calculations. It is possible to extend this model by using different charge density distributions or using Coulomb interaction energies that account for the quantum nature, to some degree. In this work we use an approach similar to extended Hückel theory for the electrode-electrode interactions which results in the same expressions for eqs 6a, 6b, and 6d, but a different expression for eq 6c, as discussed below. We then used DFT calculations to determine the values of η_*j*_ and *A*_*jk*_. These values of η_*j*_ and *A*_*jk*_ are used in subsequent HCPM simulations of the supercapacitor.

In our HCPM workflow, to determine the values of η_*j*_ and *A*_*jk*_, we carry out DFT calculations in which a Li^+^ ion is placed at different positions near the MXene to sample the charges on the MXene atoms q*(***R***) as a function of the Li^+^ ion position ***R***. We separately calculate the MXene atom charges in the absence of the Li^+^ ion, ***q***_χ_^*^, and then calculate the “induced charge” as the difference ***q***_χ=0_^*^(***R***) = q*(***R***) – ***q***_χ_^*^. For comparison, we calculate the HCPM-induced charges as ***OA***^–1^(***b***(***R***) + Δψ***d***), with the elements of ***b*** given by eq 6d, and Δψ is adjusted using the constrained charge ensemble^39^ so that the HCPM and DFT electrodes have the same total induced charge. The HCPM-induced charges will depend parametrically on η_*j*_ and *A*_*jj*_, and minimizing the difference in the values of ***q***_χ=0_^*^(***R***) from the DFT and HCPM calculations determines the optimal η_*j*_ and *A*_*jj*_ and the corresponding HCPM-induced charges.

The final charge on each electrode particle that is used in the simulation for the full system is then just the sum of the induced charges determined from the optimized η_*j*_ and *A*_*jk*_ and the constant partial charges. In this work, instead of employing the partial charges obtained from our DFT calculations to represent the electronegativities of different electrode elements (i.e., ***q***_χ_^*^), we used those from a force field developed for the simulations of the MXene-based supercapacitors.^40−43^ This is because the electrolyte-electrode non-Coulombic interaction parameters were optimized based on these force field charges, and therefore this is expected to give more accurate simulation results, while using ***q***_χ_^*^ directly from DFT calculations of MXene in vacuum would require simultaneous reparametrization of the non-Coulombic interactions. Refer to Table S1 for the partial charges for MXene atoms obtained from both our force field and DFT calculations.

#### 2.1.3 Interaction Kernel Incorporating Tunable Gaussian Widths and Hardness Offsets

As an extension of the original model discussed above, in this work, we determine the charge-dependent interaction energies between atoms in an electrode, eq 6a, based on an approach that is similar to extended Hückel theory and the chemical potential equalization method of York and Yang.^37^

In our model, hardness offsets, *f*_*j*_, are added to self-interaction terms *A*_*jj*_ (also known as the hardness, which is a measure of the resistance of a chemical species to changes in its electronic configuration^44,45^), and the interactions *A*_*jk*_ are extended following the approach used in extended Hückel theory giving
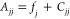
6e
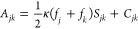
7where *C*_*jk*_ are the Coulomb terms described above and *S*_*jk*_ are overlap integrals. This approach modifies the ***A***-matrix diagonal elements in a similar manner to the Thomas–Fermi model^46^ or Hubbard U-parameter adjustment^37^ in other CPM literature, but our extended model makes corrections dependent on element and chemical environment and including their effects in interaction terms between atoms. κ is a global mixing factor, which we set to 1.75 following Hoffmann.^47^

Modeling the atoms of metallic electrodes as Gaussian charges is foundational to the constant potential method,^48^ widely recognized and utilized across numerous studies.^30,32,49−52^ Given the metallic and conductive nature of Ti_3_C_2_T_*x*_ MXene,^53,54^ we likewise model it as such in our simulations. While recognizing that Gaussian charge application to metal atoms is a simplification that glosses over some complexities—which can be otherwise captured in more detail by ab initio density functional theory^55^—this approach considerably lowers computational demands and permits simulations over extended time scales. This modeling approach, in concert with the point charge representation of the electrolyte, has proven effective in investigating the capacitive behavior of supercapacitor electrodes in many studies.^29,49,51,52,56^ Hence, in line with the CPM, we adopt a Gaussian charge distribution for MXene atoms within the HCPM MD framework. It is important to note that both HCPM and CPM simulations represent electrode charges by Gaussian charges with fluctuating magnitudes in accordance with eq 6c, which aligns with our MD framework.

To calculate the overlap integrals, we consider Gaussian functions ψ_*j*_(***r***) of width η_*j*_ centered at position ***r***_*j*_ on each atom so that
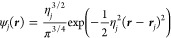
8obeying ⟨ψ_*j*_|ψ_*j*_⟩ = 1. This will give charge density distributions and Coulomb terms consistent with eqs 6b and 6c, respectively. The overlap integrals between orbitals ψ_*j*_ and ψ_*k*_ are then
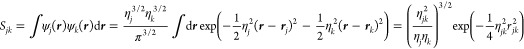
9where *r*_*jk*_ = |***r***_*j*_ – ***r***_*k*_| is the distance between the two orbital centers, and
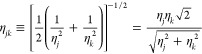
10

The Coulombic term *C*_*jk*_ can be evaluated as follows, according to eq 6c
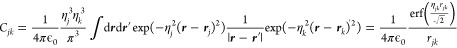
12with  which can be obtained from taking the limit *r*_*jk*_ → 0 and noting that lim_*x*→0_ (erf (*x*)/*x*) = 2/

In this approach, there are two sets of independent parameters to be determined, η_*j*_ and *A*_*jj*_ noting , and *A*_*jk*_ can be determined from eq 7. We assume that all atoms in chemically equivalent positions have the same values of η_*j*_ and *A*_*jj*_, so for the MXene system, there are 10 parameters altogether (for O, H, C, and Ti in the two different layers). These are determined by minimizing the deviation between induced charges calculated using the HCPM approach (***OA***^–1^***b***(***R***)), and those obtained using DFT calculations.

Note that for only one atom type, and setting all hardness offsets *f*_*j*_ to zero, these terms reduce to the usual CPM expressions: when η_*j*_ = η_*k*_, then η_*jk*_ = η_*j*_ = η_*k*_; as η_*k*_ → ∞ (as in the interaction between a Gaussian charge *j* and a point charge *k*), we have η_*jk*_ → η_*j*_√2; as *r*_*jk*_ → 0 (as for a Gaussian charge’s self-interaction) we have .

Qualitatively, positive values of the hardness offsets *f*_*j*_ result in more disperse charge distributions (as seen in the Salanne group’s studies of Thomas–Fermi models^46^), while negative values of *f*_*j*_ lead to more sharply localized charges (as seen from Nakano and Sato’s studies of metallicity adjustments^45^). We expect that the added *S*_*jk*_ overlap integrals have similar effects and either spread out or localize charge distributions, for positive and negative values of *f*_*j*_, respectively, although, since the exponential function in *S*_*jk*_ decays much more strongly than the function  in *C*_*jk*_, we might expect the effects of the overlap integrals to affect only nearest-neighbor charge spreads, in particular electronic charge splitting across bonds.

### 2.2 DFT Calculations of the Induced Charges on MXene

To obtain the charge density and atom-resolved charges we performed plane wave projector augmented wave density functional calculations as implemented in the VASP code.^57−60^ We used the PBE^61^ density functional approximation, a well converged 500 eV plane wave cutoff, 2 × 2 × 1 γ-point centered k-point grids, and a 12.97 × 11.11 × 40.00 Å supercell containing 16 units of the MXene, Ti_3_C_2_(OH)_2_. Calculations were carried out for this structure and for those with the addition of a Li atom in various positions. The all-electron charge density was obtained by adding the core charges to the pseudo charge density. Atomically resolved charges were obtained using the Bader scheme from the all-electron charge density.^62^

### 2.3 Fitting HCPM Coefficients from DFT Results

**Figure 1:**
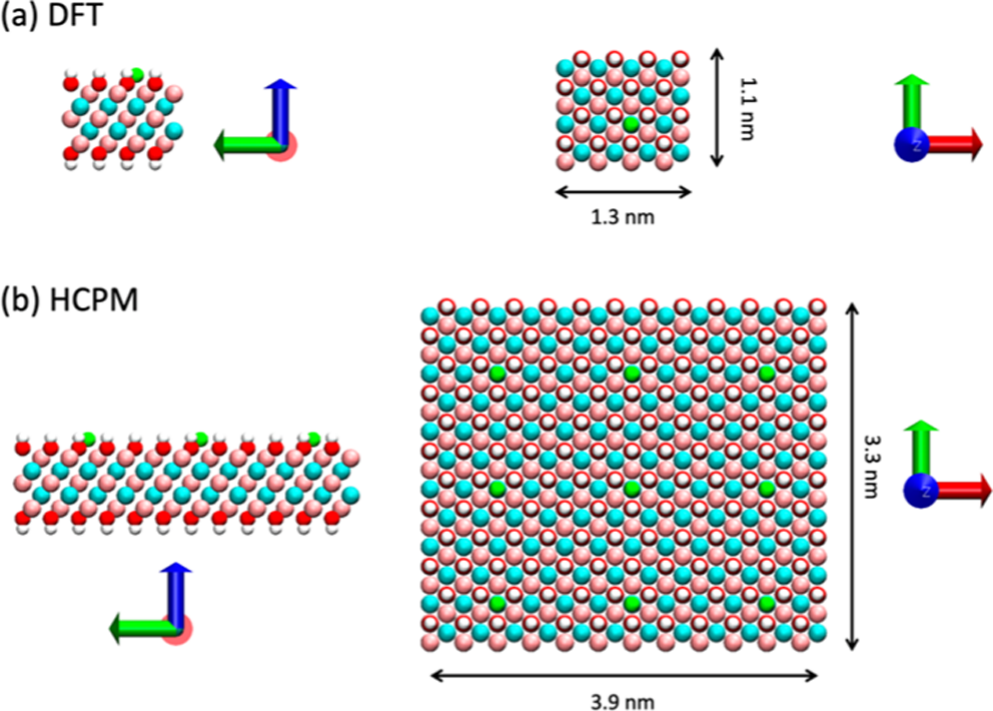
Configuration setup for (a) DFT calculations and (b) HCPM parameter fitting. The configuration utilized for DFT calculations is duplicated in the *x* and *y* directions twice to obtain the configuration for HCPM. Li^+^, white: hydrogen (H), red: oxygen (O), pink: titanium (Ti), and cyan: carbon (C).

To find the optimal values of η_*j*_ and A_*jj*_ for each type of MXene atom for use in the final HCPM simulations, we need to compare the induced electrode charge by HCPM with that obtained through DFT calculations in the presence of the Li^+^. Initially, we created a configuration with a single Li atom positioned near the MXene surface. The position of the Li atom relative to the MXene surface groups was obtained through MD simulations of the MXene and Li^+^ ions without applying any voltage (using a constant charge method). Refer to the section below for further details on the simulations. The Li^+^ ions closest to the MXene surface exhibited relatively stable behavior, resembling vibrations occurring between the MXene surface groups. By averaging the positions of these Li^+^ ions, we obtained the averaged Li atom position relative to the MXene surface groups. Subsequently, as illustrated in Figure 1a, we constructed a small system for DFT calculation, comprising a small segment of MXene and a single Li atom. The position of the Li atom relative to the MXene surface was determined by using the averaged Li^+^ ion position, and several configurations were generated by displacing Li^+^ in the *z*-direction relative to this position (vertical to the MXene surface). To perform parameter fitting for HCPM, we replicated the cell in both the *x* and *y* directions to generate a larger cell (as depicted in Figure 1b), as our HCPM calculations utilized a cutoff of 1.2 nm for both electrostatic and van der Waals interactions.

To fit the HCPM parameters, we minimized the residual sum of squares (RSS) per configuration between the induced atom charges derived from DFT and those derived from HCPM. The minimization process was achieved by tuning η_*j*_ and *A*_*jj*_ for each type of atom. Because the explicit formula for the derivatives of HCPM simulations was not analytically tractable, we utilized the derivative-free algorithm, bound optimization by quadratic approximation (BOBYQA) solver by Powell,^63^ which is well suited for solving nonlinear and nonconvex least-squares minimization problems without requiring any derivatives of the objective. This approach is also particularly well suited for our fluctuating objective function, which lacks a discernible pattern and thus cannot be effectively modeled by Gaussian process-based models such as those used in Bayesian optimization.^64,65^ Given that all systems were charge-neutral during DFT calculations, we imposed a constraint on the total charge of the electrode in the HCPM to be the opposite value of the total charge of the Li^+^ ions derived from DFT calculations. We utilized 5 different configurations for the HCPM parameter fitting, each differing in distance from the original Li^+^ position by increments of 0.1 Å (ranging from 0 to 0.4 Å) and determined the residual sum of squares (RSS) for each configuration as follows
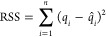
13where *n* is the total number of atoms in the MXene, *q*_*i*_ refers to the induced charge on atom *i* as determined by DFT, and *q̂*_*i*_ stands for the induced charge of atom *i* as predicted by HCPM. The averaged RSS calculated from the 5 different configurations was employed as the objective function for the derivative-free optimization. To facilitate convergence to reasonable values, we initialized the optimization with η_*j*_ = 1.979 Å^–1^ and *A_jj_* = 28.5 eV/e^2^, aligning with the parameters for the original CPM used for graphite, and constrained the η and *A* for each atom type within specific bounds, i.e., 0.1 ≤ η_*j*_ ≤ 50 Å^–1^ and 1 ≤ *A_jj_* ≤ 200 eV/e^2^. Additionally, we set κ to a fixed value of 1.75, as suggested by Hoffmann.^47^

### 2.4 Molecular Dynamics Simulation Details

**Figure 2:**
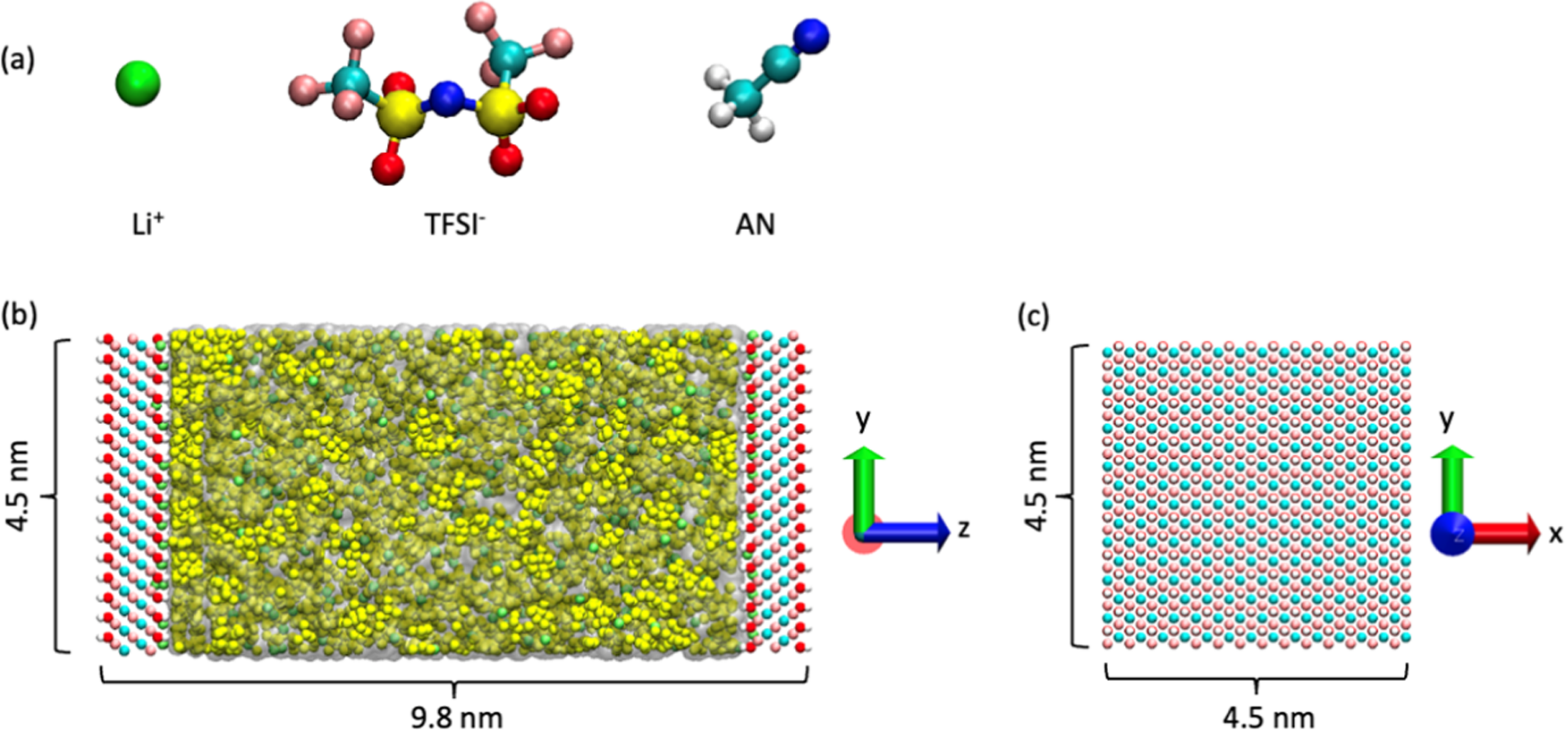
Schematic representation of the MD simulation system setup. (a) Molecular structure of the all-atom model of ions and solvent (Li-TFSI/AN) used in this study (pink: fluorine, cyan: carbon, yellow: sulfur, red: oxygen, blue: nitrogen, white: hydrogen, green: lithium). (b) Li-TFSI/AN electrolyte (green: Li^+^; yellow: TFSI^–^; transparent surface: AN) with MXene electrodes on two sides (white: hydrogen, red: oxygen, pink: titanium, cyan: carbon). (c) Top view of the MXene structure. The system is periodic only in the *x* and *y* directions.

Our simulated systems are illustrated in Figure 2, and we conducted MD simulations at 0, 1, and 2 V using the HCPM and CPM methods, as detailed in the previous section. For further comparison, we also conducted simulations using constant charge method (CCM) and with no voltage applied. In this context, “CCM” means the electrode atom charges are from the MXene force field and remain fixed throughout simulations (no induced charges on electrodes). “CPM” means that the atom charges on electrodes are fluctuated during simulations, and all electrode atoms initially have zero partial charge as the approach assumes the equivalence of all electrode atoms. “HCPM” means that all electrode atoms retain the partial charges from the MXene force field and allow additional induced and fluctuated charges on electrodes based on our modified charge response.

The MD setup procedures were similar to those reported in our previous studies.^40−42^ To model Li^+^ and AN, we used the all-atom optimized potential for liquid simulations (OPLS-AA) force field,^43^ and for TFSI^–^, we employed the CL&P force field of Canongia Lopes and Pádua.^66^ The partial charges of ions have been scaled by a factor of 0.8 to consider the effects of charge transfer and polarizability.^20,41,66,67^ The CL&P force field was designed to be compatible with the OPLS-AA force field,^43,66^ and the two force fields have been used together in many previous studies.^20,22,41,68^ For the MXene (Ti_3_C_2_(OH)_2_) force field, Coulombic (partial charges) and non-Coulombic (Lennard-Jones) parameters for the MXene (Ti_3_C_2_(OH)_2_) were used as in our previous work.^69−72^ We conducted our HCPM MD simulations using LAMMPS^73^ with the USER-CONP2 code, which has a publicly available implementation of our HCPM algorithm^74^ on GitHub. All simulations were performed under the canonical (NVT) ensemble with a time step of 2 fs, and all MXene atoms were held fixed throughout all simulations. A real-space cutoff of 1.2 nm was set for both electrostatic and van der Waals interactions, and the long-range electrostatic interactions were handled using the particle–particle particle-mesh (PPPM) method^75^ with a relative accuracy of 10^–5^. To equilibrate the systems, we initially performed 30 ns of MD simulations using CCM. For CPM and HCPM simulations, we followed this with 4 ns simulations under zero voltage and then proceeded with 40 ns of production simulations at the desired voltages. For CCM simulations, 20 ns of production simulations were performed after equilibration. The last 8 ns of these simulations were used to analyze the density and charge distributions. VMD was used for visualization.^76^

## 3 Results and Discussion

**Figure 3:**
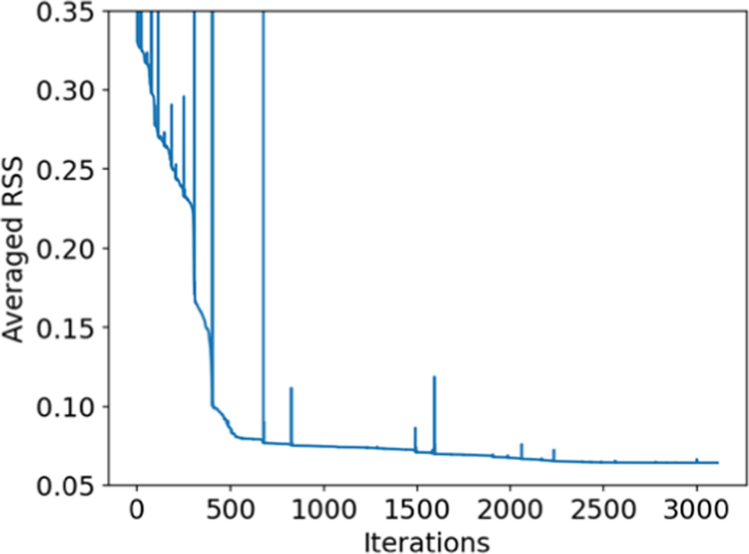
Averaged RSS per configuration as a function of optimization iterations.

Figure 3 illustrates the convergence of the averaged RSS after approximately 3000 iterations, reaching a reduction of 5 times compared with its initial value. The parameters η_*j*_ and A_*jj*_, obtained from this convergence for each atom type, which were utilized in the HCPM simulations, are provided in Table 1. The size of the Gaussian metal sites for each type of MXene atom is controlled by η_*j*_, while the self-interactions or chemical hardness is represented by *A*_*jj*_. The combined influence of these two parameters for each element governs the distribution of induced charge among atoms of the same type and different types. It is important to emphasize that the charge distribution information cannot be directly inferred from the value of a single parameter, as the charge distribution is determined collectively by the interaction of both parameters. The incorporation of these two parameters for each MXene atom type enhances the versatility of the HCPM model, allowing for easy fitting to a wide range of materials.

**Table 1:** HCPM Parameters for Ti_3_C_2_(OH)_2_ Obtained from the Derivative-Free Optimization^a^

atom type	η_*j*_ (Å^–1^)	*A_jj_* (eV/e^2^)
H	0.890	14.3
O	7.74	22.1
Ti	4.13	14.1
C	3.92	13.7
Ti’	0.950	31.7

a Ti’ denotes the Ti atoms in the innermost layer of MXene.

**Figure 4:**
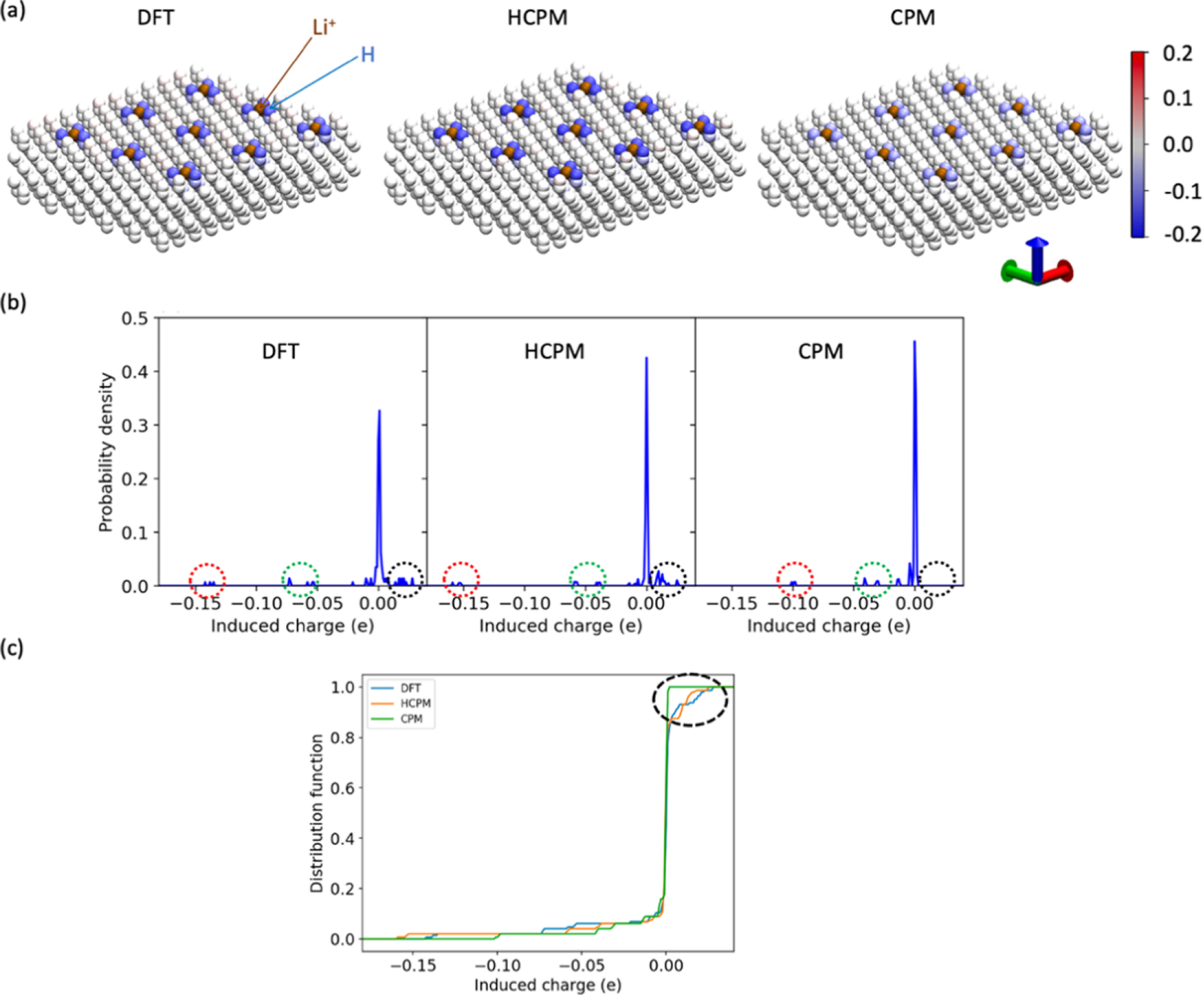
(a) Visualization of induced atom charges on MXene due to the Li^+^ ions with partial charges of +0.61*e* (brown), calculated using the methods of DFT, HCPM, and CPM. The color scale bar indicates the values of the induced charges in units of *e*. (b) Probability density functions and (c) cumulative distribution functions for induced charges on MXene by different methods. Red dashed circles: H atoms on MXene near Li^+^ ions; red dashed circles: O and Ti atoms near Li^+^ ions; black dashed circles highlight the MXene atoms with small positive induced charges present in DFT and HCPM calculations but absent in CPM calculations. The Li^+^ position relative to the MXene is from MD simulations using the CCM.

Using the optimal η_*j*_ and *A*_*jj*_ parameters and force field values for ***q***_χ_^*^ for the MXene atoms in HCPM, we determined the total charges on the electrode atoms in the presence of a Li^+^ ion. We visualized the induced charges on the MXene atoms and analyzed their probability density functions and cumulative distribution functions, as presented in Figure 4. The Li^+^ positions used in this analysis were obtained from MD simulations using CCM as mentioned in the Section 2. For information on Li^+^ positions at other locations, see the Supporting Information (SI). Notably, the HCPM-induced charges on the atoms near Li^+^ were found to be closer to the DFT result than those generated by CPM, as shown in Figure 4. While the difference between DFT and CPM is not considerable concerning the induced charge on the hydrogen atoms closest to Li^+^, the results by HCPM exhibit greater alignment with DFT. Specifically, the HCPM induced charges of ca. −0.14*e* on the hydrogen atoms near the Li^+^ atoms, and DFT induced charges of ca. −0.16, whereas CPM resulted in induced charges of ca. −0.10*e*. Moreover, for the nearest hydrogen atoms, the induced charge difference between DFT and CPM is even greater in other cases, as shown in Figure S1. For example, for the configuration where the Li^+^ is 0.4 nm above its original position, the induced charges from the DFT calculations, HCPM and CPM are ca. −0.18*e*, ca. −0.15*e*, and ca. −0.10*e*, respectively.

In addition to the H atoms surrounding Li^+^, HCPM also produced induced charges that more closely mirrored the DFT results for the nearest O (second layer) and Ti (third layer) atoms around Li^+^, as circled in green in Figure 4b. Furthermore, the distribution of induced charges differs between these methods for charge values beyond 0*e*, as illustrated in Figure 4b,c. Both HCPM and DFT methods predict small positive induced charges on a greater number of atoms compared to CPM, and these positive charges are predominantly observed near the MXene surface. The broader spread of induced charges in HCPM and DFT calculations compared to CPM indicates that MXene atoms, even those further from Li^+^, are more susceptible to Li^+^ perturbations in HCPM and DFT than in CPM. The adjusted induced charge distribution by HCPM highlights the model’s flexibility in adapting to DFT results, leading to tailored metallic properties in MXene. Our HCPM model can be expanded to other materials like conducting MOFs and MoS_2_, as well as MXenes with various terminal groups, through fine-tuning of the HCPM parameters. Additionally, the computational costs for HCPM MD and CPM MD simulations are quite similar (e.g., ∼9.0 ns/day for CPM MD versus ∼8.6 ns/day for HCPM MD in our study). This is expected as the matrix *A* is calculated prior to the simulation runs; hence, it does not incur additional computational costs during simulations. Moreover, the task of reintroducing force field charges to MXene atoms at each time step constitutes a very minor portion of the total computational cost per time step.

**Figure 5:**
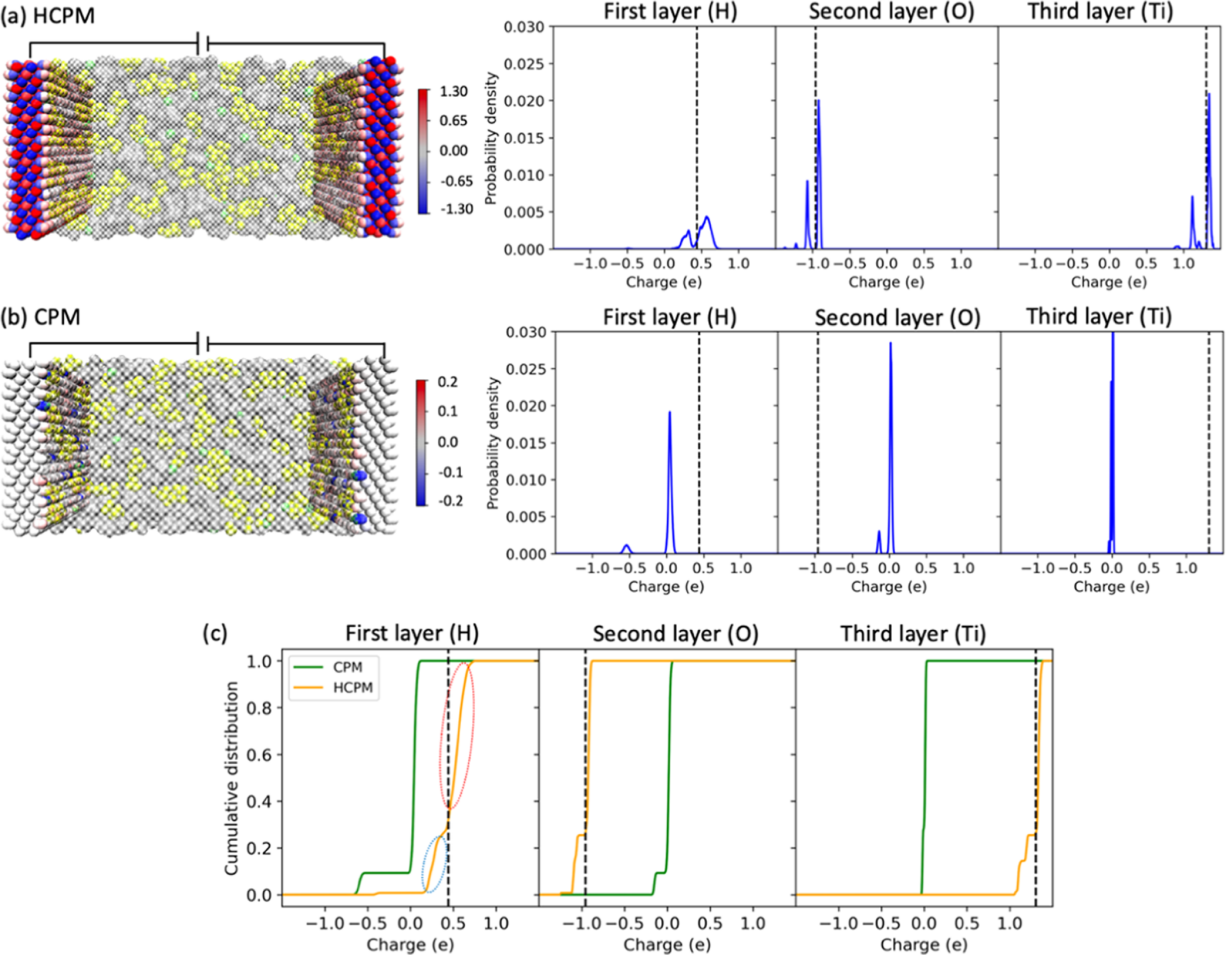
Probability density function of the atom charges on the positive electrode at 0 V, computed using HCPM (a) and CPM (b) models, along with their corresponding accumulated distribution function in (c). The visualized charges for MXene are shown on the left-hand side of (a, b), with a color scale bar indicating the values in units of *e*. Only the first three layers of MXene near the electrolyte are shown: the first layer, composed of H atoms; the second layer, composed of O atoms; and the third layer, composed of Ti atoms. The black dashed lines correspond to the force field charges of the corresponding atom types.

Although the induced charge parameters are calculated using charges in vacuo, the differences between the CPM and HCPM are found to transfer to full molecular dynamics simulations. We present this in Figure 5, showing the zero voltage CPM and HCPM probability density functions and cumulative distribution functions for the atom charges on the first three MXene layers adjacent to the electrolyte (other MXene layers are shown in Figure S2). As expected, with the CPM approach, the majority of atom charges is near zero, creating a sharp contrast with the HCPM method, where the atom charges are distributed around the force field charges. Both theoretical studies using Density Functional Theory (DFT)^77−79^ and experimental research^80,81^ indicate that MXene atoms have significant nonzero partial charge, while the CPM MD approach incorrectly predicts that most atoms inside the electrode are neutral because of the assumption that without any external influence, the partial charges in the electrode will be zero. However, HCPM maintains significant partial charges, in agreement with both theory and experiment.

Our analysis further shows that the H layer of MXene adjacent to the electrolyte exhibits two peaks in the charge distribution from the CPM simulations, as illustrated in Figure 5b. While the majority of charges are close to 0.1*e*, some are around −0.6*e*. The large negative charge of −0.6*e* on the hydrogen atoms in CPM simulations is attributed to the assumption that in CPM that the electrodes are ideal metals and all atoms in the electrodes are of the same type for calculation of charges,^31,33^ as well as the fact that some Li^+^ ions are positioned close to these hydrogen atoms.

Surface functionalization plays a crucial role in determining the electronic properties of MXene. For example, the introduction of −OH terminal groups can cause the pristine MXene (Ti_3_C_2_) to transition from a metallic to a semiconducting material.^82−85^ The attenuation of MXene metallicity resulting from the passivation of the −OH functional groups conflicts with the assumption made by the CPM, which assumes that all atoms in the electrodes are ideal metallic atoms including the terminal groups. Without this assumption, the charge of −0.6*e* on the hydrogen atoms observed in CPM simulations is less likely to occur, while the HCPM-generated charge distribution is much more realistic. As shown in Figure 5c, and noting the H force field charge is 0.44*e*, the charge distribution on the H atoms within MXene using HCPM is close to the value but wider than that using CPM. The broader charge distribution could be associated with our modified charge response as indicated in Figure 4b,c where the induced charge distribution extends beyond 0*e* in HCPM and DFT calculations. Similar to CPM simulations, the H layer in HCPM simulations displays two charge peaks, as well. The emergence of one charge peak at approximately 0.25*e* is primarily a result of induction effects from the nearest Li^+^ ions to the H layers, whereas the second, slightly larger peak that is closer to 0.5*e* is mainly due to induction effects from other atoms and ions.

**Figure 6:**
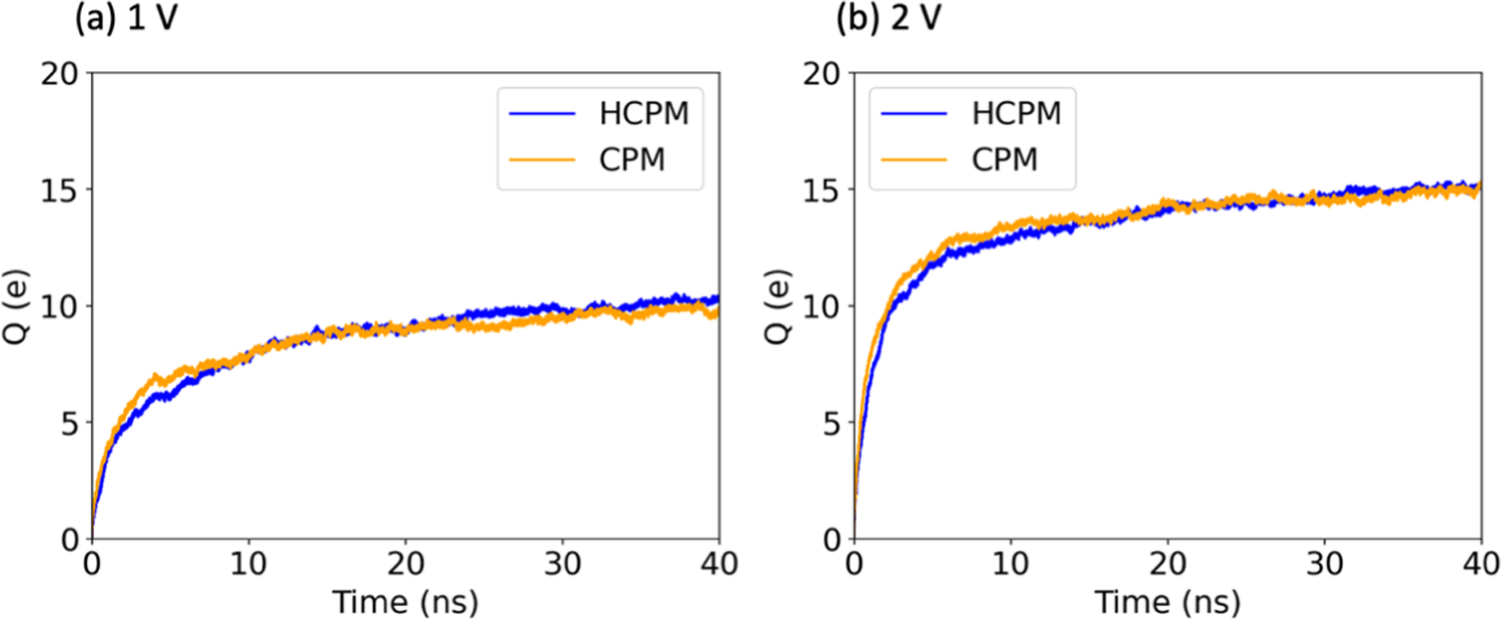
Time evolution of the total accumulated charge (*Q*) on the positive electrode using CPM and HCPM under (a) 1 and (b) 2 V. The yellow and blue lines represent the potential difference applied by CPM and HCPM, respectively.

**Figure 7:**
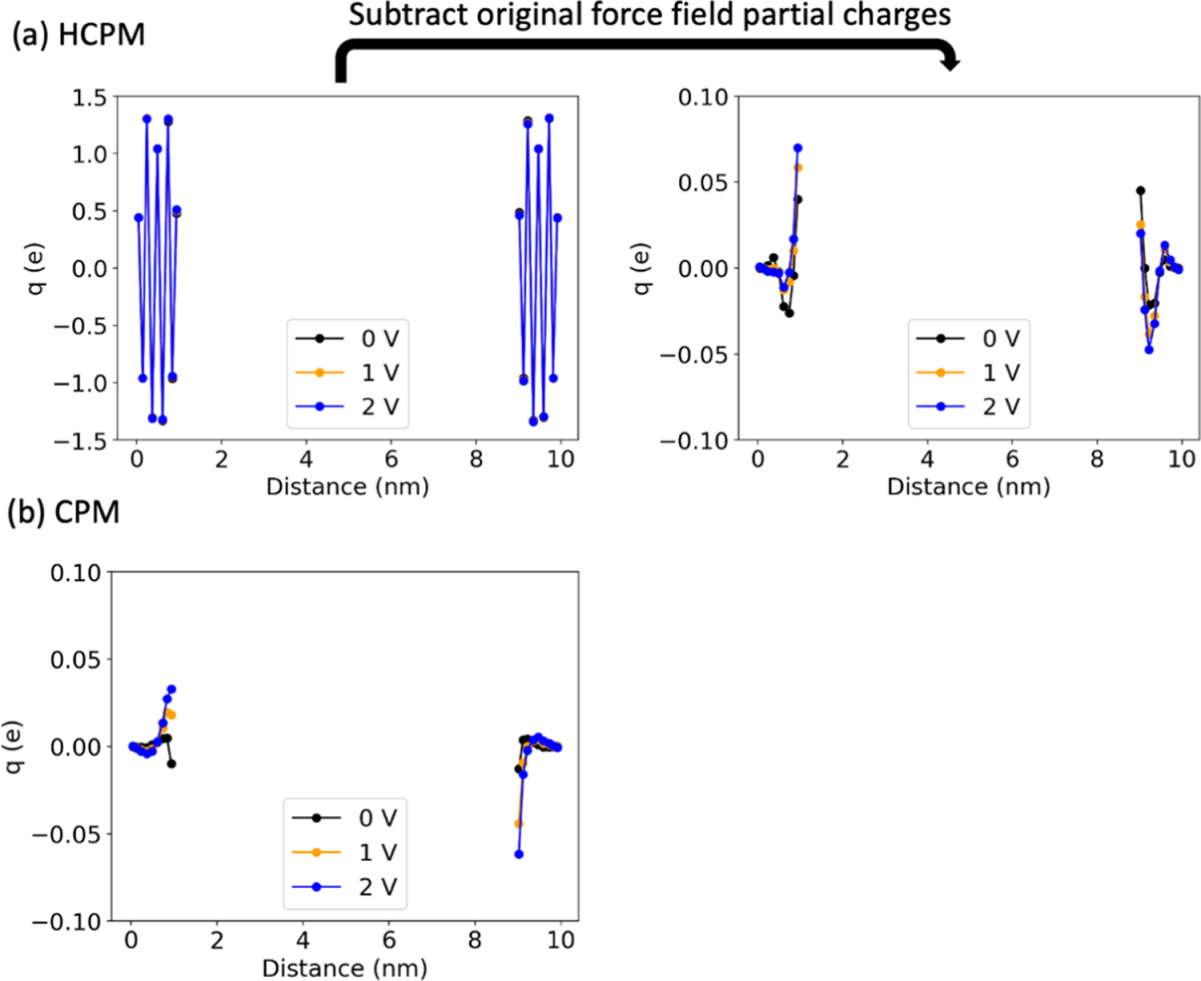
Averaged atom charge for each MXene layer on both sides as a function of distance in the *z* direction by (a) HCPM (left: total averaged atom charges per MXene layer; right: induced averaged atom charges per MXene layer) and (b) CPM.

Figure 6 depicts the time evolution of the total charge accumulated on the positive electrode under 1 and 2 V using HCPM and CPM. The charge storage obtained by both methods is similar, indicating that the combined effects of different MXene atom electronegativities and charge response abilities have little impact on the charging dynamics and total charge storage of planar MXene systems. To further investigate how each layer of MXene contributes to charge storage, we examined the induced charges on MXene. Figure 7a (left) shows the total average charge on the atoms in each layer during HCPM simulations, and Figure 7a (right) shows the induced charges of each layer of MXene obtained by subtracting the force field atom charges from the total atom charges. Comparing the results of Figure 7a (right) with those of Figure 7b shows that although the magnitude of induced charges is similar for HCPM and CPM, the trends in the induced charge on each MXene layer differ. The variation in induced charge between HCPM and CPM is primarily on the first few layers of the MXene surface, with the hydrogen atoms adjacent to the electrolyte exhibiting the most significant difference. For example, at 0 V, the hydrogen layer in HCPM has an induced charge of +0.04*e*, while the surface hydrogen atoms in CPM have an induced charge of −0.01*e*. At a positive electrode potential of 2 V, HCPM induces almost double the average charge on the surface hydrogen compared to CPM (+0.07*e* for HCPM and +0.03*e* for CPM), while on the negative electrode, HCPM induces a low positive charge (+0.02*e*) on the hydrogen layer, in contrast to the relatively larger negative charge by CPM (−0.06*e*).

Experimental results from electrochemical in situ X-ray absorption near edge structure spectroscopy (XANES) and electron energy loss spectroscopy (EELS) show that the oxidation state of MXene atoms weakly changes upon charging.^77,80,86^ This agrees with the findings from our HCPM simulations, as shown in Figure 7a, where we observed that the average charge of each layer of atoms remains relatively stable at various voltages. The minor shift in MXene’s oxidation state suggests that using CCM seems to be an acceptable approach for MD simulations involving MXene. However, although the CCM has been used in some MXene studies to investigate the effects of solvent^78^ and ionic liquids^87^ on charge storage, the CCM has been found to produce unrealistic molecular dynamics in simulations compared to CPM,^24,30^ studying the charging dynamics of MXene electrodes using CCM is unfeasible. Ideally, simulations should employ a constant potential method, such as HCPM, which permits charge fluctuation for conductive materials and takes into account the variable nature of heteroatomic electrodes.

**Figure 8:**
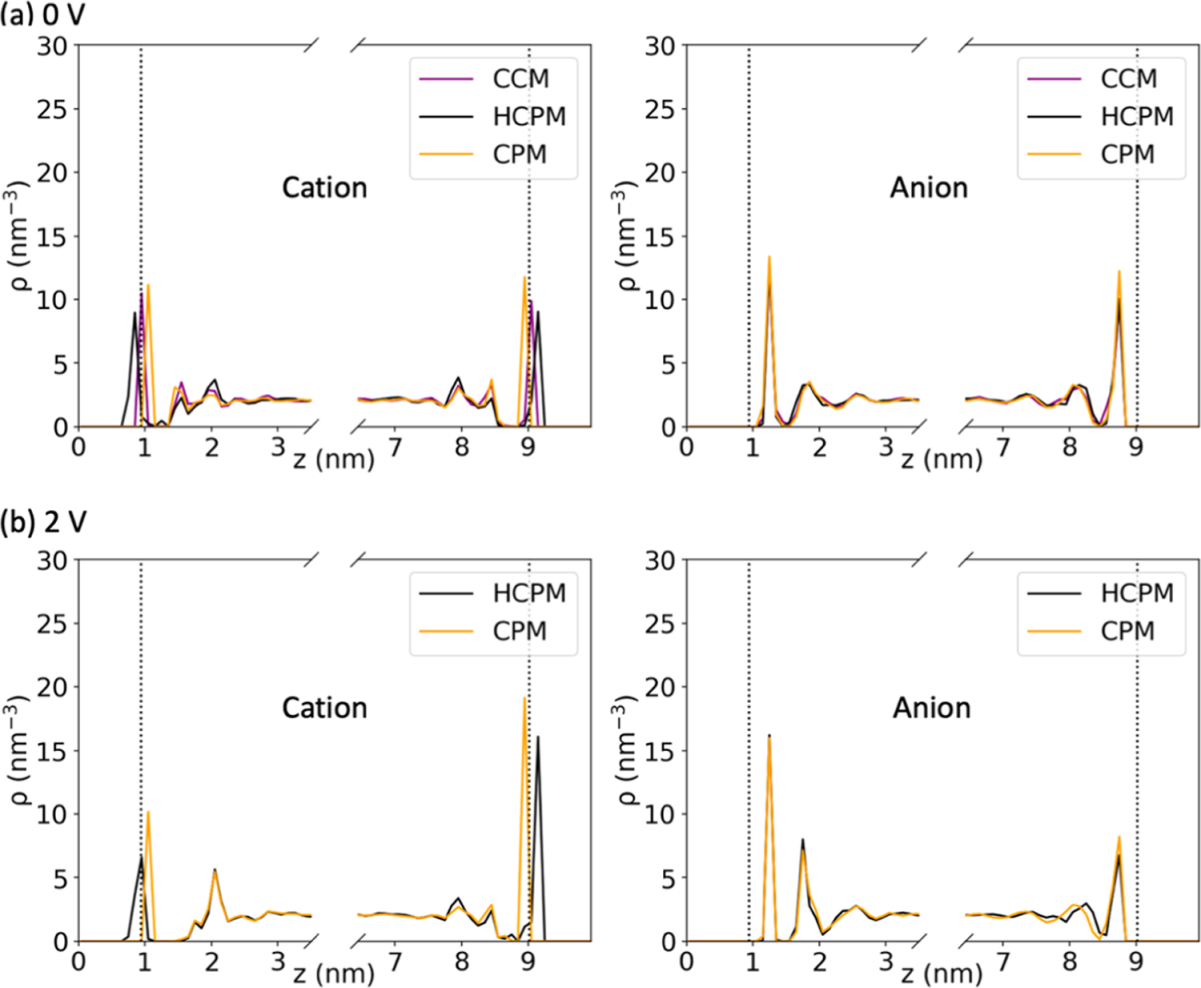
Ion number density distribution along the *z* direction by HCPM, CPM, or CCM under (a) 0 and (b) 2 V. The dashed gray lines represent the boundaries of the electrode.

To investigate the effects of the CPM and HCPM simulations on the structures of liquid ions in proximity to the MXene surface, we computed the center-of-mass (COM) ion density distribution along the *z*-axis under 0 and 2 V, as presented in Figure 8. Our findings indicate that the anion density distributions were comparable for the HCPM and CPM simulations at 0 and 2 V and for CCM at 0 V. As expected, the anions were attracted toward the positive electrode and repelled from the negative electrodes at 2 V, while the cations showed the opposite trend, with higher density on the negative electrode than on the positive electrode.

Contrary to the similar behavior of the anions for the different methods, the cations were found to be closer to the MXene electrodes in HCPM compared to their positions in the CPM at both 0 and 2 V, as shown in Figure 8. At 0 V, the first cation peak in the HCPM simulations exhibits the closest proximity to the MXene electrodes, followed by CCM, and then CPM. The increased cation–MXene interactions observed in CCM, as compared to CPM, can be attributed to the incorporation of the original partial charge of MXene atoms. Furthermore, the enhanced cation–MXene interactions observed in HCPM, as compared to CCM, result from cations inducing charge on the MXene, while it retains its original partial charge from the force field, thereby amplifying these interactions in HCPM.

**Figure 9:**
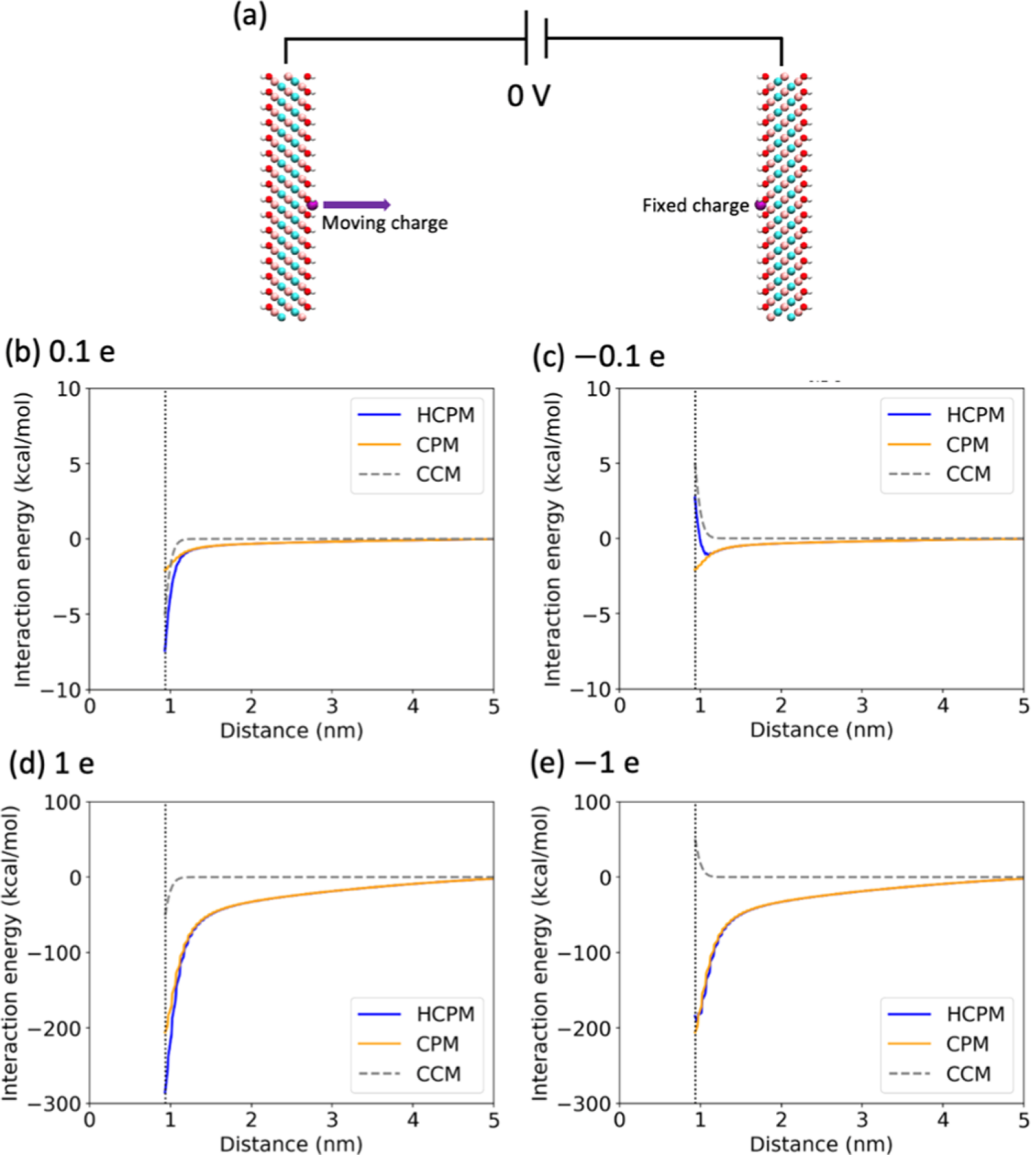
Electrostatic interaction energy between the positive electrode and the left-side point charge (purple) as the charge point moves toward the center along the *z*-axis. (a) Visualization of the slab system. The left-side point charges are (b) 0.1, (c) −0.1, (d) 1.0*e*, and (e) −1.0*e*. The fixed charge (purple) is anchored on the right side of each system, and its value equals the opposite value of the corresponding point charge on the left side to keep the system neutral. The potential difference is set at 0 V. The dashed gray line is the right boundary of the positive electrode.

To further understand the underlying factors contributing to the variation in ion density distributions, we analyzed the electrostatic interaction energy between a point charge and the positive electrode at 0 V, following a similar approach as in the study conducted by Bi and Salanne,^29^ as shown in Figure 9. The point charge was moved away from the MXene sheet toward the center of the cell along the *z*-axis (Figure 9a). Our results showed that the interaction energy calculated by the HCPM and CPM methods was similar when the point charge was far from the MXene surface but significantly different as it approached MXene closely. However, it should be noted that this simplified analysis does not fully represent the complex interactions present in simulation systems, considering the presence of many particles. Nonetheless, compared to CPM, the HCPM results in a stronger attraction between the point positive charge and the MXene when the point charge is near MXene, which suggests an enhanced interaction between MXene and cations in HCPM simulations. It is worth noting that the interaction curve found using CCM deviated from HCPM and CPM even at longer distances from MXene, but no corresponding difference is present in the ion density profile at longer distances. This suggests that the electrostatic interactions are effectively screened by charged particles in the system.

As we discussed above, the inclusion of electronegativity effects and adjusted charge response is crucial for heteroatomic electrodes, since these factors shape the charging behavior of different electrode elements and control the liquid ion structure. The electronegativity of heteroatomic electrodes can also markedly influence the frictional forces between electrodes and electrolytes, and by extension, the charging mechanism.^29,88,89^ Therefore, we recommend the use of HCPM for any studies involving charging dynamics and charge storage in heteroatomic supercapacitor electrodes. Nonetheless, for future research, we highly recommend creating more general HCPM parameters through the fitting of extensive DFT calculations across different systems with the goal of developing a unified HCPM model suitable for diverse applications. The use of HCPM adds little to the computational cost and is more realistic in the sense that it takes into account the charge on each MXene atom in the presence of an applied potential.

Moving forward, it is also important to recognize the limitations inherent in our model. Our HCPM shares certain limitations with conventional CPM, adhering to similar simplified assumptions used in classical molecular dynamics, such as assigning fixed values to electrolyte atom charges and disregarding charge transfer and polarizability of the electrolyte during simulations. Despite these simplifications, our HCPM, compared to conventional CPM, achieves a significant increase in physically interpretable accuracy by incorporating new per-element parameters and electronegativities.

## 4 Conclusions

In this study, we have developed the heteroatomic constant potential method (HCPM) for studying heteroatomic MXene supercapacitors, adding the capability of studying general heteroatomic systems to conventional CPM. The HCPM method provides an approach to consider the atom electronegativity and adjust the metallic properties of different atom types. Using a derivative-free optimization method, we were able to tune the charge response of different atoms in our HCPM parameters to resemble the results of the DFT simulations. By incorporating electronegativity and modifying the charge response of different MXene atoms, our HCPM method produces a more accurate charge distribution on MXene electrodes with an altered liquid cation structure. We chose to use force field partial charges and charges from a Bader analysis of the DFT calculations to determine the HCPM parameters. However, we note that other suitable models for the Coulomb interaction energy could be used in the HCPM implementation. Further studies are required to determine which give the best representations of the electrostatic potential in the electrode material considered.

We believe that the HCPM method has great potential for further studies of heteroatomic supercapacitors and can contribute significantly to understanding the fundamental mechanisms of energy storage in these systems. Overall, this work stands as a significant theoretical advancement in the field of heteroatomic supercapacitors, unveiling new avenues for the development of high-performance energy storage devices utilizing HCPM simulations.

## Supporting Information Available

Partial charges of MXene atoms from the force field and DFT calculations; induced charges on the MXene with varying Li^+^ positions (PDF)
