# Supplementary material for: Development of Heteroatomic Constant Potential Method with Application to MXene-Based Supercapacitors
Source: J Chem Theory Comput. 2024 Jan 11;20(2):651–64. doi: 10.1021/acs.jctc.3c00940 (PMC10809414; doi:10.1021/acs.jctc.3c00940)
Supplement: Supplementary file 1 — ct3c00940_si_001.pdf [file ct3c00940_si_001.pdf]

Development of heteroatomic constant potential method for Mxene-based supercapacitors

Xiaobo Lin[1,2,†], Shern R. Tee[3,†], Paul R. C. Kent[4], Debra J. Searles[3,5], Peter T. Cummings[1,2,6,]*

[1]*Multiscale Modeling and Simulation Center, Vanderbilt University, Nashville, TN, USA*
[2]*Department of Chemical and Biomolecular Engineering, Vanderbilt University, Nashville, TN, USA*
[3]*Australian Institute for Bioengineering and Nanotechnology, The University of Queensland, Brisbane, QLD, AU*
[4]*Computational Sciences and Engineering Division, Oak Ridge National Laboratory, Oak Ridge, TN, USA*
[5]*School of Chemistry and Molecular Biosciences, The University of Queensland, Brisbane, QLD, AU*
[6]*School of Engineering and Physical Sciences, Heriot-Watt University, Edinburgh, SCT, UK*
[†]*These authors contribute equally to this work.*

Table S1. Partial charges of MXene atoms used in the force field (FF) and those derived from our DFT calculations (without Li atoms). Ti' denotes the Ti atoms in the innermost layer of MXene.

Atom type	q_i in FF (e)	q_i in DFT (e)
H	0.4400	0.4776
O	-0.9597	-1.3122
Ti	1.3054	1.7473
C	-1.3092	-1.7485
Ti’	1.0435	1.6698

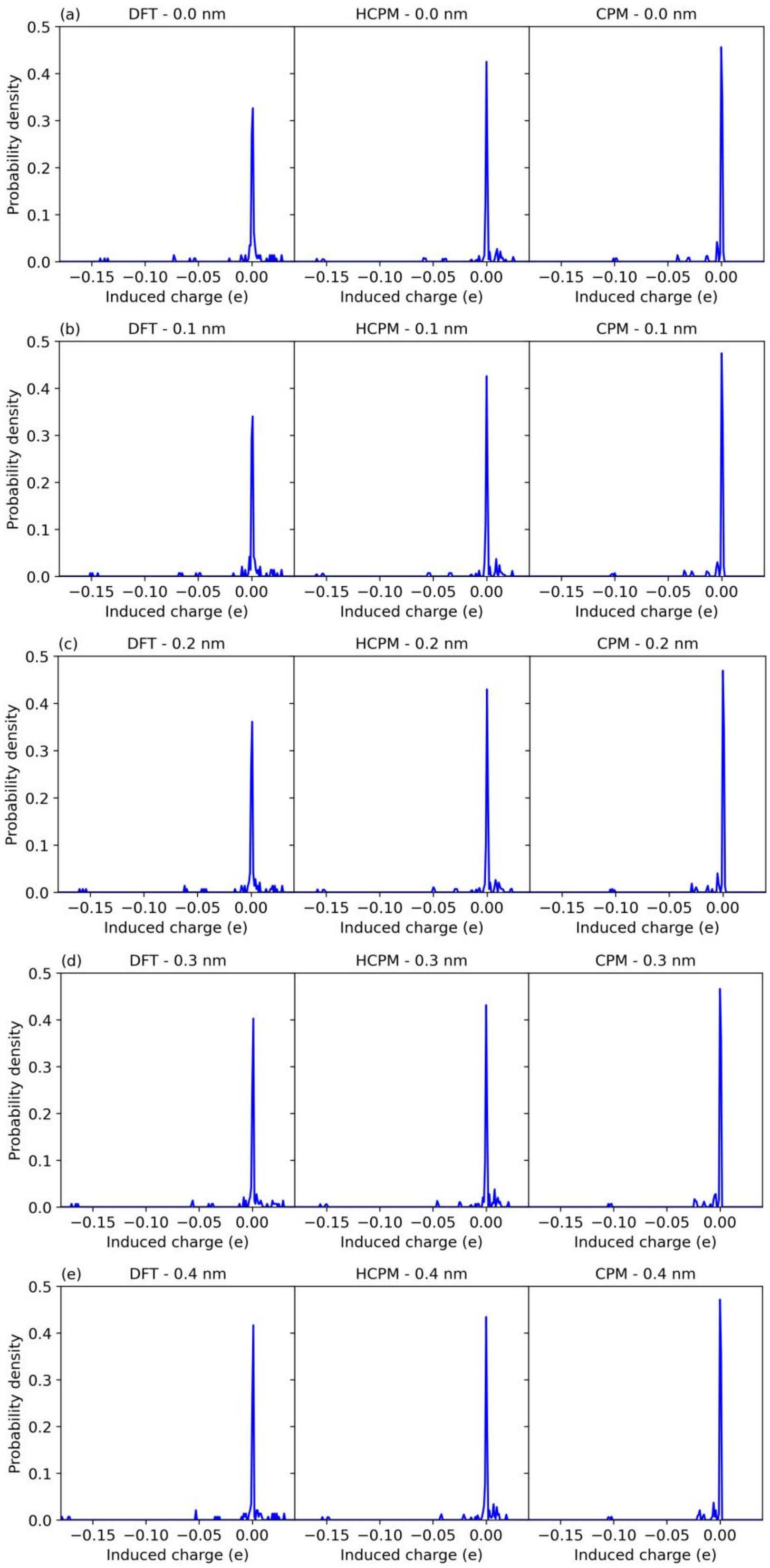

(a) DFT - 0.0 nm
HCPM - 0.0 nm
CPM - 0.0 nm
(b) DFT - 0.1 nm
HCPM - 0.1 nm
CPM - 0.1 nm
(c) DFT - 0.2 nm
HCPM - 0.2 nm
CPM - 0.2 nm
(d) DFT - 0.3 nm
HCPM - 0.3 nm
CPM - 0.3 nm
(e) DFT - 0.4 nm
HCPM - 0.4 nm
CPM - 0.4 nm
Probability density
Induced charge (e)

Figure S1. Probability density distribution of induced charges on the MXene by DFT, HCPM, and CPM with varying Li^+ positions located (a) 0.0 nm, (b) 0.1 nm, (c) 0.2 nm, (d) 0.3 nm, and (e) 0.4 nm away from the initial Li^+ position (vertically relative to the MXene).

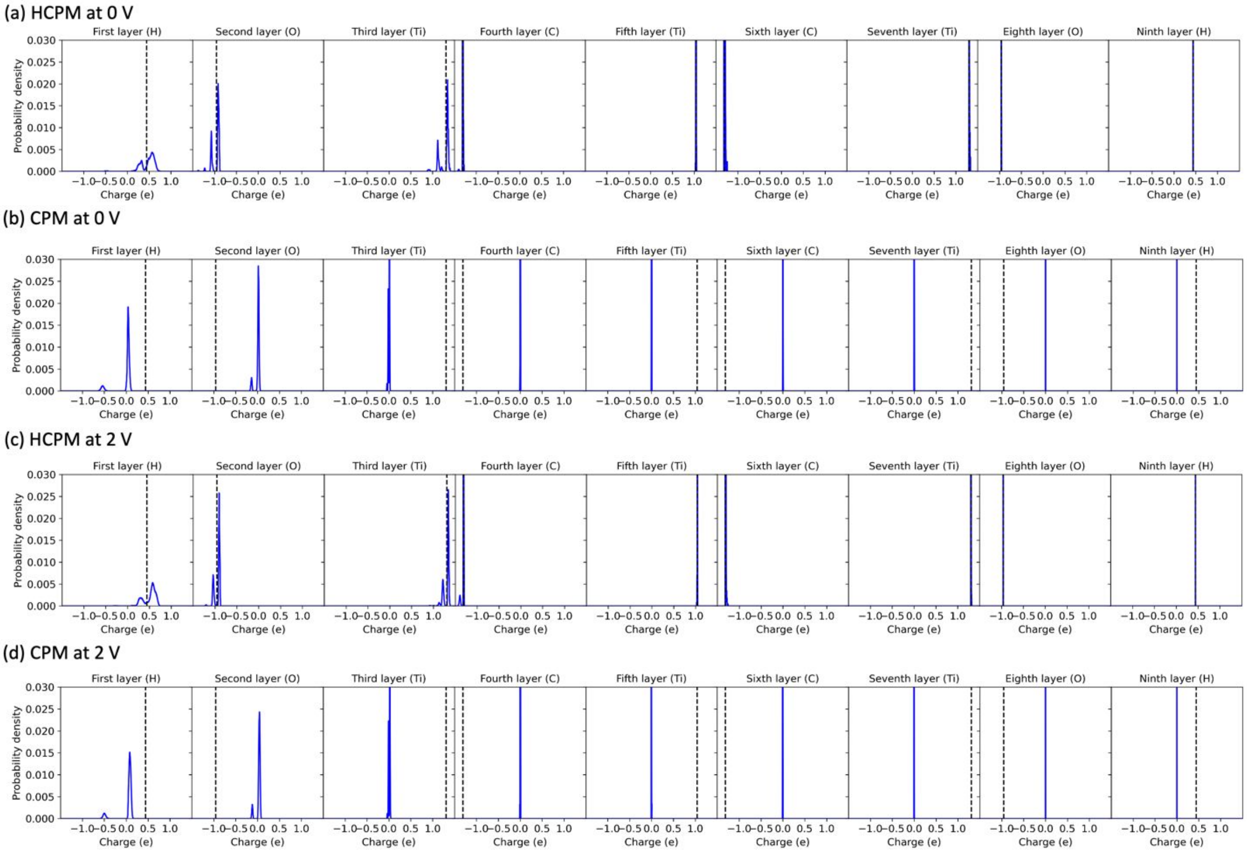

Figure S2. Distribution of the average atom charges on the positive electrode at 0 and 2 V, computed using HCPM and CPM simulations. The plots from left to right indicate increasing distance from the MXene-electrolyte interface. The dashed vertical lines represent the atom charges in the force field.
